# Isolated Periosteal Avulsion Fracture of the Teres Major in a Rugby Player

**DOI:** 10.5334/jbsr.1608

**Published:** 2018-07-31

**Authors:** Julie Desimpel, Filip Vanhoenacker

**Affiliations:** 1 AZ Sint-Maarten Mechelen/Duffel and University (Hospital) Antwerp/Ghent, BE

**Keywords:** Periosteal avulsion fracture, teres major, radiography, CT, MRI

## Abstract

We describe a case of an isolated periosteal avulsion fracture of the teres major tendon. A 29-year-old rugby player presented following a direct hit with his opponent followed by a fall on the ground. Clinical examination showed a limited and painful abduction of his left arm. MR-arthrography showed no capsulolabral lesions but revealed an extra-articular lesion at the origin of the teres major tendon. Follow-up radiography and CT showed progressive ossification of the lesion. To the best of our knowledge, this is the first case of a distal avulsion fracture of the teres major tendon reported in the imaging literature.

## Introduction

Rugby players may present with a wide variety of shoulder lesions. However, an isolated lesion of the teres major is rare. They predominantly occur at the musculotendinous junction. To the best of our knowledge, an isolated tendon-bone avulsion has not been reported yet in the imaging literature. The aim of this case report is to give an overview of the imaging characteristics of this rare lesion.

## Case Report

**Figure 1:**
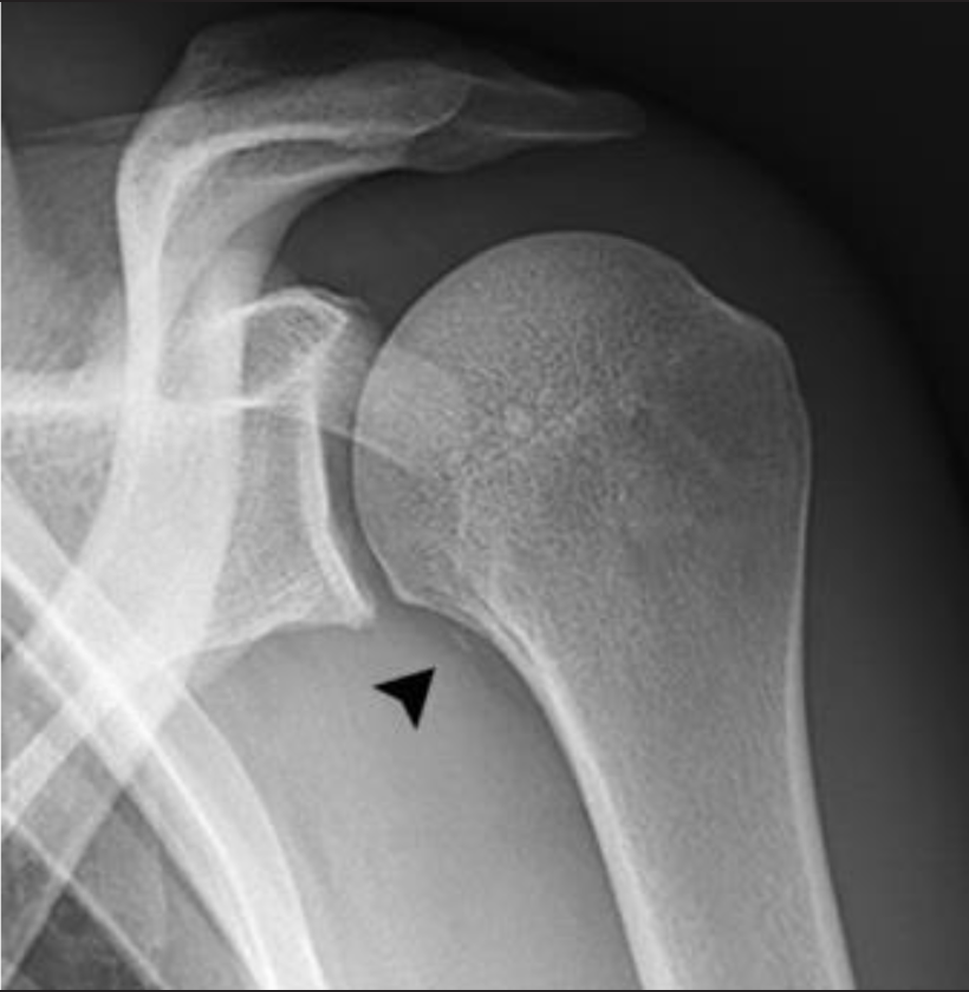
A 29-year-old rugby player presenting after shoulder trauma. Standard radiography of the left shoulder. A small fleck of bone (black arrowhead) is seen medial to the proximal humerus.

**Figure 2:**
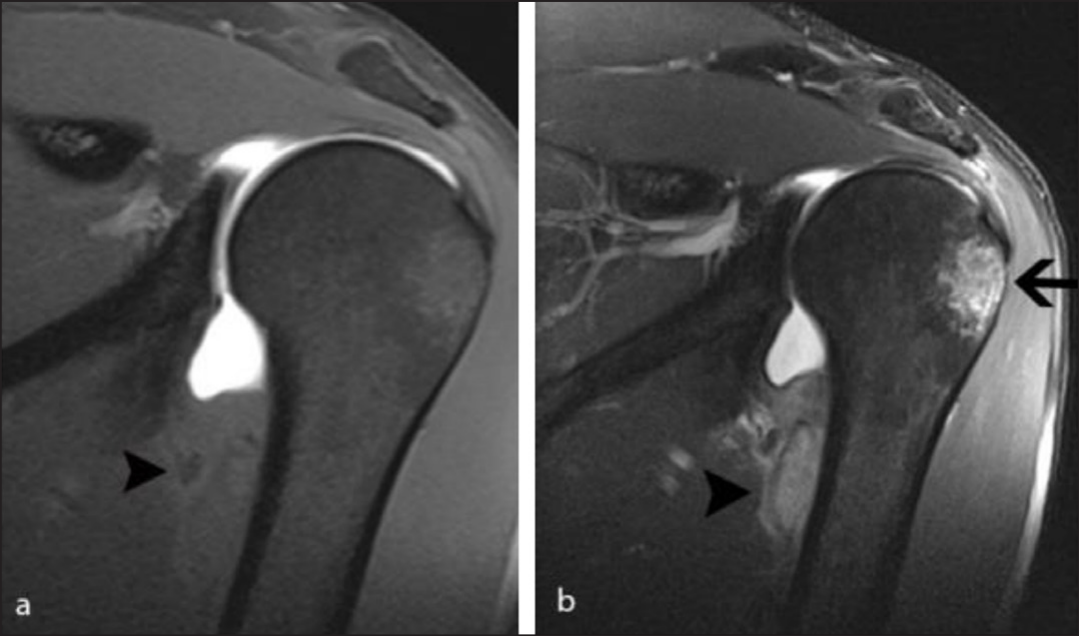
MR arthrography of the left shoulder performed four weeks after initial injury. **a)** On the coronal FS T1-WI, there is an extra-articular lesion with slightly inhomogeneous signal intensity (black arrowhead) at the medial side of the humeral diaphysis. **b)** The lesion (black arrowhead) is better seen on the FS T2-WI. Note also the posttraumatic bone marrow edema (black arrow) at the superolateral aspect of the humeral head.

**Figure 3:**
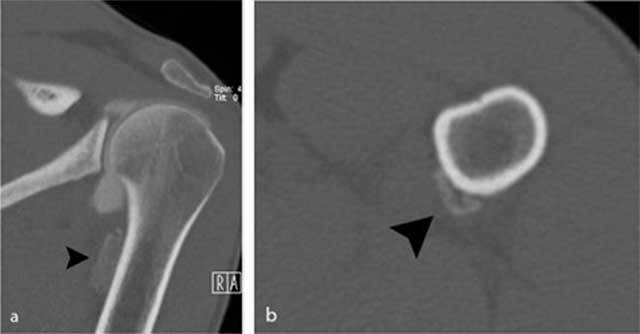
Additional CT images of the proximal humerus. **a)** Oblique coronal reformatted images and **b)** axial reformatted images reveal the presence of an extra-articular calcification (black arrowhead) near the insertion of the teres major tendon.

**Figure 4:**
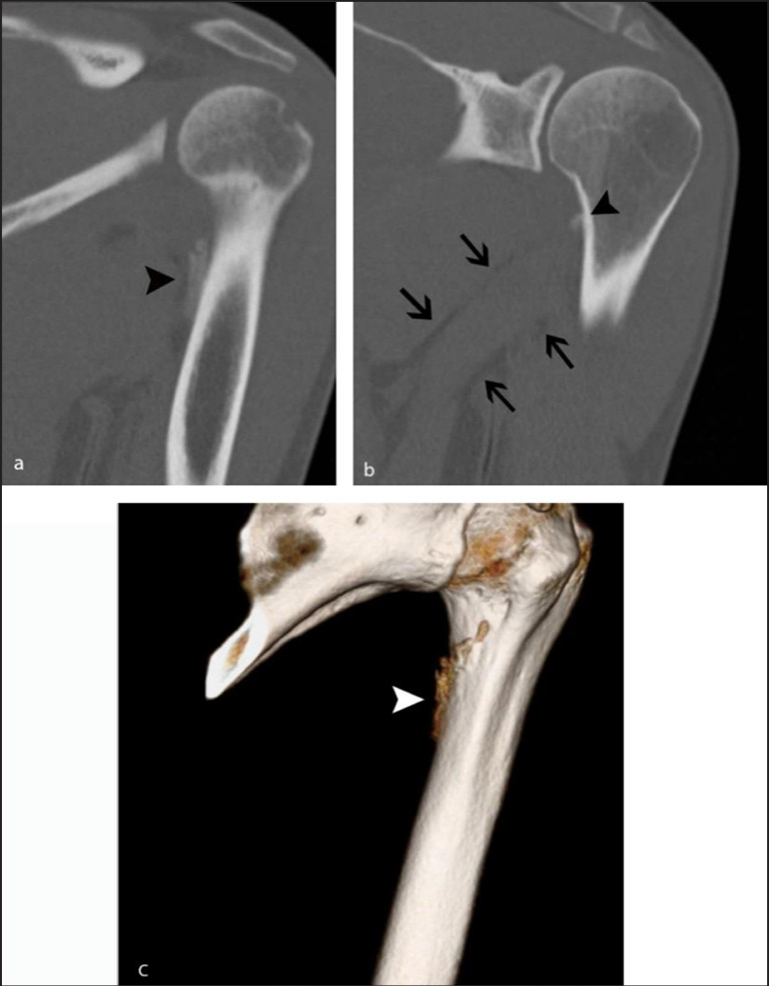
Follow-up CT five weeks later. **a)** Oblique coronal reformatted image of the proximal humerus shows a slightly more dense aspect of the periphery of the calcification (black arrowhead). **b)** MIP reconstructions (1.5 mm) clearly show that the calcification is located at the medial crest of the lesser tuberosity (black arrowhead). Note also the perimuscular fat pads surrounding the teres major muscle (black arrows). **c)** Volume Rendering Technique (VRT) image revealing irregular contour (white arrowhead) near the medial crest of the lesser tuberosity.

A 29-year-old rugby player presented with pain at the medial side of the left upper arm after a direct hit on the left shoulder by a player of the opponent team followed by sudden sense of posterior displacement of the upper arm. Clinical examination revealed difficulty to abduct the left upper arm and focal pain on palpation at the posterior axillary fold. A small fleck of bone adjacent to the medial humeral diaphysis was seen on the plain radiography (Figure 1). MR arthrography four weeks following the initial trauma revealed an inhomogeneous extra-articular lesion at the humeral insertion of the teres major tendon. Fat-suppressed (FS)-T2 WI showed posttraumatic bone marrow edema at the superolateral aspect of the humerus, which could be attributed to the direct impact on the shoulder (Figure 2). CT following MR revealed peripheral calcification in keeping with posttraumatic calcification surrounding the stripped periosteum at the insertion of the teres major tendon on the medial crest of the lesser tubercle (Figure 3). Follow-up imaging five weeks after the MR arthrography showed a subtle density increase at the periphery of the calcification (Figure 4).

**Figure 5:**
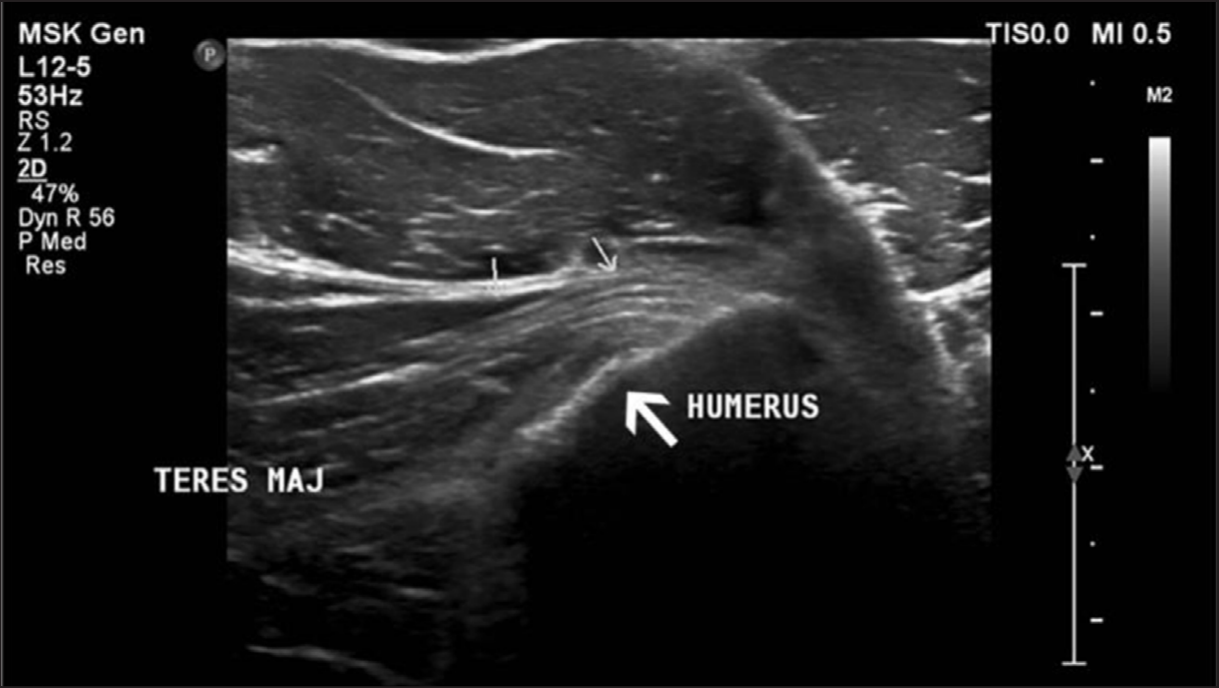
Ultrasound imaging (axial image) reveals an irregular delineation (large white arrow) of the teres major insertion at the medial aspect of the humerus posteromedial to the pectoralis major tendon (small arrows).

Ultrasound showed irregularity of the tendon insertion without discontinuity (Figure 5). The patient returned to play four months after a conservative treatment consisting of sport restriction and intensive physiotherapy.

## Discussion

An isolated periosteal avulsion fracture of the teres major is a rare entity. Although tears at the musculotendinous junction have been reported rarely [1], we found no reports of a teres major tear at its distal insertion.

**Figure 6:**
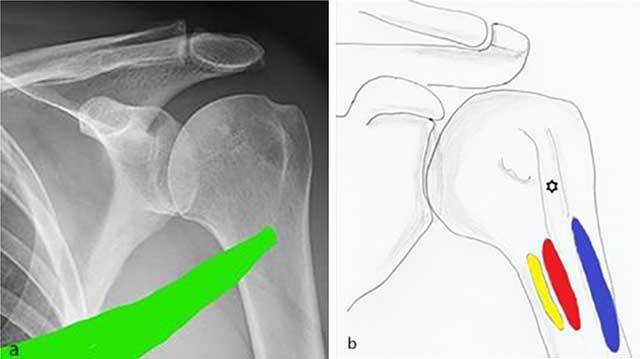
**a)** Spiral anatomical course of the teres major which may explain shearing forces leading to the periosteal stripping is demonstrated. **b)** Anatomical drawing showing the close anatomical relationship of the insertion of the teres major (yellow), latissimus dorsi (red) and pectoralis major (blue) tendon.

The teres major originates at the inferior angle of the posterior border of the scapula. It runs anterolaterally upwards to insert near the medial crest of the lesser tubercle (Figure 6). The main function of the teres major includes internal rotation, unresisted adduction and extension of the arm. Together with the latissimus dorsi muscle it has a role in ball acceleration during pitching [2].

The spiral course of the teres major generates rotational forces (Figure 6) [3]. As a consequence, trauma may result in a broad-based periosteal stripping lesion, which will curl up and progressively calcify during the follow-up of the lesion. The mechanism is comparable with medial femoral epicondylar periosteal stripping in Pelligrini-Stieda or with periosteal stripping occurring in common adductor insertion avulsion of the thigh (thigh splint) [4,5]. Due to its close anatomical relationship with the tendinous insertion of the latissimus dorsi, an avulsion fracture of both tendons may co-exist (Figure 6) [6]. Teres major tears are predominantly seen on imaging at the distal musculotendinous junction (MTJ). Generally, MTJ lesions are divided in three grades. A grade 1 tear shows feathery edema around the MTJ. A partial disruption is categorized as grade 2 lesion whilst a complete disruption is a grade 3 lesion [7]. Imaging findings of a tear at the tendon bone insertion has – to the best of our knowledge – not been reported yet.

Standard radiography in the acute phase may reveal a small subtle fleck of bone at the periosteal insertion of the teres major. This should not be misinterpreted as a bony Bankart lesion, as the lesion is located slightly more inferiorly. CT may be useful to diagnose a subtle avulsion and for follow-up showing progressive peripheral calcification and ossification and ultimately fusion with the underlying cortex [4]. Although MR (arthrography) is currently a routine imaging tool for evaluation of shoulder trauma in sports trauma, it is important to analyze the images beyond the margin of the joint and scrutinize for extra-articular lesions [8]. Therefore, at least one imaging sequence with a large field of view should be used.

Ultrasound provides dynamic evaluation of the tendon mobility but a full evaluation of the teres major muscle and tendon is hampered due to its relative deep position [9].

Similar bony fragments can be seen in case of avulsion of the latissimus dorsi or pectoralis major given the similar insertion. Other differential diagnosis includes calcific tendinitis or subperiosteal haematoma.

Standard treatment care for MTJ lesions is based on the PRICE principle (Protection, Rest, Ice, Compression, Elevation) with a favorable outcome. Patients are able to return to play after an average treatment duration of four months. Surgical treatment should only be considered in overhead athletes if conservative therapy fails. Erickson et al. concluded that all patients were able to return to pre-operative athlete activities with minimal residual pain [10]. In our patient, PRICE was implemented with a favorable outcome.

## Conclusion

In conclusion, an isolated periosteal avulsion fracture of the teres major is rare and may be challenging, as the subtle osseous avulsion on plain films may be confused with a Bankart lesion, if the extra-articular location is not considered. MRI should be analyzed beyond the borders of the joint.
